# Validation of putative reference genes for gene expression studies in human hepatocellular carcinoma using real-time quantitative RT-PCR

**DOI:** 10.1186/1471-2407-8-350

**Published:** 2008-11-27

**Authors:** Vito R Cicinnati, Qingli Shen, Georgios C Sotiropoulos, Arnold Radtke, Guido Gerken, Susanne Beckebaum

**Affiliations:** 1 Department of Gastroenterology and Hepatology, University Hospital Essen, University of Duisburg-Essen, Essen, Germany; 2 Department of General, Visceral and Transplantation Surgery, University Hospital Essen, University of Duisburg-Essen, Essen, Germany

## Abstract

**Background:**

Reference genes, which are often referred to as housekeeping genes are frequently used to normalize mRNA levels between different samples in quantitative reverse transcription polymerase chain reaction (qRT-PCR). The selection of reference genes is critical for gene expression studies because the expression of these genes may vary among tissues or cells and may change under certain circumstances. Here, a systematic evaluation of six putative reference genes for gene expression studies in human hepatocellular carcinoma (HCC) is presented.

**Methods:**

Six genes, beta-2-microglobulin (*B2M*), glyceraldehyde-3-phosphate dehydrogenase (*GAPDH*), hydroxymethyl-bilane synthase (*HMBS*), hypoxanthine phosphoribosyl-transferase 1 (*HPRT1*), succinate dehydrogenase complex, subunit A (*SDHA*) and ubiquitin C (*UBC*), with distinct functional characteristics and expression patterns were evaluated by qRT-PCR. Inhibitory substances in RNA samples were quantitatively assessed and controlled using an external RNA control. The stability of selected reference genes was analyzed using both *geNorm *and *NormFinder *software.

**Results:**

*HMBS *and *GAPDH *were identified as the optimal reference genes for normalizing gene expression data between paired tumoral and adjacent non-tumoral tissues derived from patients with HCC. *HMBS, GAPDH *and *UBC *were identified to be suitable for the normalization of gene expression data among tumor tissues; whereas the combination of *HMBS, B2M*, *SDHA *and *GAPDH *was suitable for normalizing gene expression data among five liver cancer cell lines, namely Hep3B, HepG2, HuH7, SK-HEP-1 and SNU-182. The determined gene stability was increased after exclusion of RNA samples containing relatively higher inhibitory substances.

**Conclusion:**

Of six genes studied, *HMBS *was found to be the single best reference gene for gene expression studies in HCC. The appropriate choice of combination of more than one reference gene to improve qRT-PCR accuracy depends on the kind of liver tissues or cells under investigation. Quantitative assessment and control of qRT-PCR inhibitors using an external RNA control can reduce the variation of qRT-PCR assay and facilitate the evaluation of gene stability. Our results may facilitate the choice of reference genes for expression studies in HCC.

## Background

Real-time quantitative reverse transcription (qRT)-polymerase chain reaction (PCR) is a rapid, sensitive and reliable method for gene expression studies. It is inherently an indirect method of measurement, and variabilities exist in the various steps of the qRT-PCR which may lead to severe misinterpretation of the results. The latter may be due to different amounts and quality of starting material, variable enzymatic efficiencies (i.e. efficiency of retrotranscription from RNA to complementary DNA (cDNA), and PCR efficiency) between samples and runs, operator errors, and differences between tissues or cells in overall transcriptional activity [1,2]. Thus, proper normalization strategy is necessary for reliable quantitative information to be extracted from this variable system.

Various strategies have been explored in an attempt to normalize these variations, and it is generally accepted that gene-expression levels should be normalized by carefully selected and stably expressed reference genes [2-4]. The success of this procedure is highly dependent on the choice of the appropriate reference genes. An ideal reference gene should be unaffected by the experimental conditions and should have low variation in gene expression. Otherwise, the detection of small changes becomes unfeasible, producing results that may be entirely incorrect [5]. Several reports have provided the evidence that the expression of most commonly used reference genes varies among tissues or cells and may also change under certain environmental circumstances [6-11]. For instance, a recent report indicated the de-regulation of common reference genes in hepatocellular carcinoma (HCC) arising from hepatitis C virus (HCV) infected liver [12]. Therefore, it is critical to perform preliminary evaluation studies, aimed at identifying the most stably expressed reference genes in individual tissues and distinct circumstances for each single experiment.

HCC is the sixth most frequently diagnosed cancer and the third most common cause of cancer mortality in the world [13], but the molecular mechanisms of hepatocarcinogenesis, including gene expression variation, are not well understood. Therefore, the number of studies assessing global gene expression profiles of HCC has increased exponentially in recent years [14-20], and the identification of optimal reference genes is necessary for correct gene expression profiling of HCC. In the current study, we validated the stability of six putative reference genes in liver cancer cell lines, tumoral tissues and adjacent non-tumoral tissues from 20 HCC patients. Two algorithms based on different strategies, *geNorm *and *NormFinder*, were used for data analysis.

## Methods

### Primer design

Primers for RT-PCR assays of *HMBS *and *UBC *were designed using Primer Express v2.0 (Applied Biosystems, Foster City, California, USA). Primer sequences for *B2M*, *GAPDH*, *HPRT1 *and *SDHA *were obtained from the real-time PCR primer and probe database (RTPrimerDB) [21]. Special attention was given to primer length, annealing temperature, base composition and 3'-end stability. To ensure optimal DNA polymerization efficiency, amplicon length ranged between 69 and 113 bp. Exon and intron boundaries were determined aligning primers with corresponding human genomic sequences downloaded from GenBank . RT-PCR primers for each gene were located on different exons or directly spanning exon-exon boundaries of the genomic sequence to minimize amplification from any contaminating genomic DNA that may remain following DNase treatment. To ensure that gene transcripts used as standards display a similar secondary structure with native RNA, primers for cloning were designed to have binding sites located at least 20 nucleotides external to the binding sites of primers for assays. The sequences of primers for cloning and the length of amplicons are shown in Supplementary table 1 in additional file 1.

### Samples

Human liver cancer cell lines Hep3B, HepG2, SK-HEP-1 and SNU-182 were obtained from the American Type Culture Collection (ATCC, Manassas, USA). Human HCC cell line HuH7 was a kind gift from Dr. Brigitte Pützer. Hep3B, HepG2, HUH7 and SK-HEP-1 were grown in DMEM medium (high glucose, with glutamine and sodium pyruvate) supplemented with 10% (v/v) heat-inactivated fetal bovine serum (FBS), 100 U/ml penicillin, 100 μg/ml streptomycin (all from PAA Laboratories Pasching, Austria), and 0.1 mM GIBCO™ MEM Non Essential Amino Acids (Invitrogen Corporation, Carlsbad, CA). SNU-182 was grown in RPMI 1640 medium containing 1 mM sodium pyruvate, 2 mM L-glutamine, 4500 mg/L glucose, 10 mM HEPES, and 1500 mg/L sodium bicarbonate, supplemented with 10% (v/v) heat-inactivated FBS, 100 U/ml penicillin and 100 μg/ml streptomycin (all from PAA Laboratories). Stock cultures were maintained at 37°C in a humidified, 5% CO_2 _incubator and were routinely passaged at 3 to 4 day intervals. Tumoral and non-tumoral liver tissues were obtained from 20 patients with HCC undergoing liver resection. Most of those were paired tumoral and adjacent non-tumoral tissues. All tissue samples were snap frozen in liquid nitrogen and stored till use at -80°C. The study was approved by the local ethics committee and all patients provided written informed consent. Detailed information regarding cell lines, patients and tissue samples is shown in Supplementary tables 2 and 3 in additional file 1.

### Total RNA isolation and characterization

Total RNA was extracted using the miRNeasy Mini Kit (Qiagen, Hilden, Germany), according to the manufacturer's instructions. Genomic DNA was eliminated by a DNase-on-column treatment with RNase-free DNase set (Qiagen). The purity of extracted RNA was assessed by measuring the optical density (OD) at wavelengths of 230 nm, 260 nm and 280 nm using a quartz cuvette with Eppendorf Biophotometer (Eppendorf, Hamburg, Germany). Absorbance ratio at 260/280 nm and 260/230 nm was used to assess the purity of the RNA samples. The integrity and quantity of extracted RNA were assessed by evaluating the capillary electrophoresis trace with Agilent 2100 bioanalyzer (Agilent Technologies, Waldbronn, Germany). The integrity of the samples was determined by RNA integrity number (RIN) algorithm, which assigns a score from 10 to 1, where level 10 represents a completely intact RNA, and level 1 represents a highly degraded RNA [22]. Total RNA from cell lines was extracted at the exponential growing phase. Liver tissue samples were disrupted and homogenized in QIAzol lysis reagent with TissueRuptor (Qiagen) for total RNA isolation.

### Construction of absolute standard curves

*In vitro *transcripts of each gene were generated as standards. RT-PCR products of each gene were purified with Wizard^® ^SV Gel and PCR Clean-Up System (Promega Corporation, Madison, WI, USA), and then cloned with PGEM^®^-T easy vector system (Promega), according to the manufacturer's instructions. Plasmid DNA was isolated and purified with Wizard^® ^*plus *SV Minipreps DNA purification system (Promega). The orientation and sequence of the inserts were confirmed by restriction endonuclease digestion with EcoRI (Roche Molecular Biochemicals, Mannheim, Germany) followed by agarose gel electrophoresis with ethidium bromide staining, and gene sequencing with BigDye^® ^Terminator v1.1 Cycle Sequencing Kit (Applied Biosystems, Foster City, CA, USA) using the T7 Promoter Primer (Promega). Bacterial clones with correct inserts were cultured again for isolation and purification of plasmid DNA with PureYield™ Plasmid Midiprep System (Promega). Plasmid DNA was linearized with restriction endonuclease SaI I (Roche) or NcoI (Promega) and transcribed into RNA with MEGAscript^® ^T7 or Sp6 kit (Ambion, Austin, TX, USA). Transcripts were purified with MEGAclear™ kit (Ambion) and plasmid DNA was eliminated by DNase treatment before and after the purification of the transcripts with TURBO DNase I (Ambion) and TURBO DNA-*free*™ kit (Ambion) respectively. DNase treated samples were desalted with DNase inactivation reagent included in TURBO DNA-*free*™ kit. The quantity and quality of the transcripts were evaluated with Eppendorf Biophotometer (Eppendorf), denatured agarose gel electrophoresis and Agilent 2100 bioanalyzer (Agilent). Five points of 10-fold serial dilution of each transcript were used to build the standard curve for each gene. The copy number range of each standard curve was set according to the approximate expression level of the individual gene determined by preliminary experiments and encompassed the copy number range of the genes in the samples. The copy numbers of the standards are shown in Supplementary table 4 in additional file 1.

### qRT-PCR inhibitor detection

qRT-PCR inhibitor was detected in each sample and standard using Alien^® ^QRT-PCR Inhibitor Alert (Stratagene, La Lolla, CA, USA), according to the manufacturer's instructions. Briefly, each sample was spiked with 10^5^copies of alien RNA transcript, an *in-vitro *transcribed RNA being non-homologous to the sequences currently available in GenBank. Increases in the threshold cycle (Ct) value for amplification of alien RNA in samples were compared with alien RNA alone and calculated to determine the presence of inhibitory substances in the samples [23].

### Real-time quantitative RT-PCR

Real-time quantitative RT-PCR (qRT-PCR) was performed with the SYBR Green QuantiTect RT-PCR Kit (Qiagen), according to the manufacturer's instructions. For each sample, 20 ng total RNA was used in the assay and all the genes were tested with the same panel of RNA samples. RT negative reactions were performed for standards and sample RNA. All standards and samples were run in duplicate on 96-well reaction plates with the iQ™5 Multicolor Real-Time PCR Detection System (Bio-Rad, Hercules, CA, USA). Reactions were prepared in a total volume of 25 μl containing 20 ng RNA in 5 μl volume, 1.25 μl of each 10 μM primer (500 nM), 12.5 μl of SYBR Green master mix, 0.25 μl of RT mix and 4.75 μl RNase/DNase-free sterile water. No template controls were run for each master mix and primer pair. After performing preliminary gradient real-time RT-PCR assays, the optimal annealing temperature for all the primer pairs resulted to be 56.4°C. At this temperature the lowest Ct value was generated as well as a sharp melting peak, with no amplification of non-specific products or primer-dimer artifacts (see Additional file 2, 3, 4, 5, 6, 7). The conditions of reverse transcription and primer concentrations were set according to the recommendations in the manufacturer's instructions and confirmed by preliminary experiments which generated correlation coefficient (R^2^) of each standard curve above 0.99. The cycle conditions were as follows: reverse transcription at 50°C for 30 min, DNA polymerase activation and RT enzyme inactivation at 95°C for 15 min, followed by 40 cycles of denaturation at 94°C for 15 s, primer annealing at 56.4°C for 30 s, elongation at 72°C for 30 s. The data collection step was performed at a temperature that was 3°C lower than the melting temperature of each amplicon and higher than that of primer-dimers of each primer pair. This cycle was followed by a melting curve analysis, ranging from 55°C to 95°C, with temperature increasing steps of 0.5°C every 10 s. Baseline and threshold values were automatically determined for all plates using the Bio-Rad iQ5 Software 2.0. The obtained data were analyzed using *geNorm *(version 3.5, written by Vandesompele J. et al) and *NormFinder *software (written by Andersen C.L. et al).

## Results and discussion

Six putative reference genes from different abundance and functional classes were selected for evaluation: *B2M*, *GAPDH*, *HMBS*, *HPRT1*, *SDHA*, and *UBC *(Table 1). Ribosome RNAs, like 28s rRNA and 18s rRNA were not included in this study, because imbalance between rRNA and mRNA fractions has been observed and rRNA transcription is affected by biological factors and drugs [24-27]. Gene sequence information deposited in GenBank under the Accession Numbers was used to design RT-PCR primers (Table 2). The qRT-PCR efficiency for each single primer pair was determined in real-time RT-PCR using serial 10-fold dilutions of *in vitro *transcripts of each gene. Expression patterns of the six reference genes have been subsequently evaluated in different sample groups.

**Table 1:** Putative reference genes evaluated.

**Gene**	**GenBank accession number**	**Name**	**Function**	**Genomic localization**	**Gene Aliases**	**IMAGE**
*B2M*	NM_004048	Beta-2-microglobulin	Beta-chain of major histocompatibility complex class I molecules	15q21-q22		51940
*GAPDH*	NM_002046	Glyceraldehyde-3-phosphate dehydrogenase	Oxidoreductase in glycolysis and gluconeogenesis	12p13	G3PD	510510
*HMBS*	NM_000190.3	Hydroxymethyl-bilane synthase	Heme synthesis, porphyrin metabolism	11q23		245564
*HPRT1*	NM_000194.1	Hypoxanthine phosphoribosyl-transferase 1	Purine synthesis in salvage pathway	Xq26	HGPRT	345845
*SDHA*	NM_004168.2	Succinate dehydrogenase complex, subunit A	Electron transporter in the TCA cycle and respiratory chain	5p15	FP; SDH2; SDHF	375812
*UBC*	NM_021009.3	Ubiquitin C	Protein degradation	12q24		510582

**Table 2:** Details of primers and amplicons for the 6 evaluated genes.

**Gene**	**Primer**	**Sequence (5' → 3')**	**Genomic Position**	**Amplicon Length**	**correlation coefficients (R^2^)**	**qRT-PCR Efficiency (%)**
*B2M*	Forward	CTCCGTGGCCTTAGCTGTG	1st Exon	69 bp	0.998	90.1
	Reverse	TTTGGAGTACGCTGGATAGCCT	1st/2nd exon			
*GAPDH*	Forward	TGCACCACCAACTGCTTAGC	7th Exon	87 bp	0.997	92
	Reverse	GGCATGGACTGTGGTCATGAG	7th/8th exon			
*HMBS*	Forward	TGCAACGGCGGAAGAAAA	1st/2nd Exon	113 bp	0.998	91.0
	Reverse	ACGAGGCTTTCAATGTTGCC	3rd exon			
*HPRT1*	Forward	TGACACTGGCAAAACAATGCA	6th Exon	94 bp	0.997	94.9
	Reverse	GGTCCTTTTCACCAGCAAGCT	6th/7th exon			
*SDHA*	Forward	TGGGAACAAGAGGGCATCTG	2nd Exon	86 bp	0.996	87.8
	Reverse	CCACCACTGCATCAAATTCATG	3rd exon			
*UBC*	Forward	CGGTGAACGCCGATGATTAT	1st Exon	124 bp	0.994	88.4
	Reverse	ATCTGCATTGTCAAGTGACGA	1st/2nd exon			

### RNA quantity and quality determination

All RNA samples were examined for their purity, integrity and concentration. The absorbance ratio at 260/280 nm of all the samples were all over 2.0, except for one sample with a ratio of 1.85, indicating all the samples were free from proteins potentially accumulating during the RNA extraction procedure. The absorbance ratios at 260/230 nm were 0.43 ~2.23, indicating possible contamination of some samples with carbohydrates, peptides, phenols or aromatic compounds [28]. Although samples with a low OD 260/230 ratio may interfere with downstream process like RT-PCR [28], only a few studies using RT-PCR methods have taken the OD 260/230 ratio into consideration when evaluating RNA sample purity. In addition, there is no generally accepted cutoff value of OD 260/230 ratio that allows researchers to determine the suitability of a sample to be used in qRT-PCR. Thus, RNA samples were additionally subjected to qRT-PCR inhibitor detection for the mining of the relationship between OD 260/230 ratio and qRT-PCR inhibition. All RNA samples were determined to be intact or to have mild loss of integrity (RIN > 6.5) using the RIN algorithm [22]. A RIN higher than 5 was recommended as good total RNA quality and a RIN higher than 8 as perfect total RNA quality for downstream qRT-PCR application [29]. Therefore, the integrity of all RNA samples was suitable for the use in our experiments. The absorbance ratio at 260/280 nm, 260/230 nm and RIN are shown in Supplementary table 5 in additional file 1.

### Presence of qRT-PCR inhibitors in purified RNA samples

**Figure 1:**
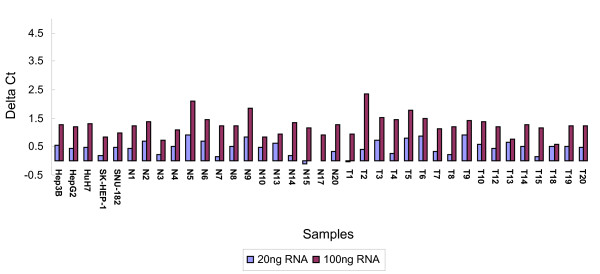
**QRT-PCR inhibitor detection in all samples**. ΔCt for amplification of alien RNA in samples of 100 ng and 20 ng RNA respectively were compared with that of Alien RNA alone. Each sample was spiked with 10^5 ^copies of alien RNA transcripts.

Increases of the Ct value (ΔCt), for amplification of alien RNA in 100 ng samples compared with alien RNA alone ranged between 0.16~2.37. When the samples were diluted 5-fold, increases of the Ct value, ranged between -0.14~0.92, indicating that the inhibition of qRT-PCR by inhibitors was significantly reduced by simply diluting the experimental RNA samples (Figure 1), but was not reduced to an acceptable level in some samples.

Pearson's correlation analysis between the OD 260/230 ratios and ΔCt values resulted in a Pearson's r of -0.2420, r^2 ^of 0.0586, and a p value of 0.175, indicating no statistically significant linear relationship between the two variables (Table 3). Data shown here suggest that an alien RNA control, but not the OD 260/230 ratio, is an direct and specific way for the detection of qRT-PCR inhibitors in RNA samples. Thus, the selection of samples in this study was based on the quantitative assessment of qRT-PCR inhibitors using an alien control RNA. Samples with a ΔCt value higher than the 75th percentile of all the samples, namely 0.675, were excluded from the analyses for the determination of suitable reference genes. For paired tissues, both tissues were excluded from the analysis if the ΔCt value of one of them exceeded the cutoff value.

**Table 3:** Pearson's correlation analysis between the existence of qRT-PCR inhibitors and OD 260/230.

	**median**	**75th**	**Mean**	**STD**	**Normality test**	**Pearson's correlation coefficient**
ΔCt*	0,49	0.675	0.47	0.26	P ≥ 0.20	r = -0.2420 (r^2 ^= 0.0586), p = 0.175
OD 260/230	1.49	1.965	1.49	0.48	P ≥ 0.15	

Both of the variables were determined to be approximately normally distributed prior to the calculation of Pearson's correlation coefficient. * Amplification of alien RNA in 20 ng RNA samples compared with alien RNA alone; OD260/230: the ratio of optical density (OD) at wavelength of 260 nm to that at 230 nm. 75th: 75th percentile.

There is a variety of inhibitors potentially affecting the efficiency of qRT-PCR reactions which may be co-purified with RNA samples, depending on the source of starting material and the methods of extraction. Tissues are among the common sample types known to contain inhibitors like collagen [30]. Inhibitors from experimental reagents include phenol, ethanol, guanidine, EDTA and others [23]. Thus, it is important to assess the existence of qRT-PCR inhibitors in RNA samples from tissues prior to the qRT-PCR assay.

We found no significant correlation between the OD 260/230 ratio and the level of qRT-PCR inhibitors in RNA samples. Moreover, it has been shown that some inhibitors may directly bind to nucleotides [31]. Therefore, whether a modification of the RNA extraction procedure or a repurification of RNA samples will reduce the level of qRT-PCR inhibitors needs further validation.

### Presence of contaminating DNA in standards and purified RNA samples

RT negative reactions for standards confirmed completely removal of plasmid DNA. There were PCR amplifications in RT negative reactions of B2M and GAPDH in two samples with Ct values much lower than those of RT positive reactions of the same samples, indicating a negligible copy number of amplified contaminating DNA (see additional file 8). There were no PCR amplifications of the other four genes in RT negative reactions for the samples.

### Standard curves and qRT-PCR efficiency of each primer pair

The qRT-PCR efficiency of each primer pair was determined by serial dilutions of homologous external RNA of each gene. Homologous RNA standards are most suitable for two reasons [32]: (i) RNA standards (as opposed to DNA) must be used to control the variability during the RT step; (ii) homologous external RNA standards share the same primer binding site as the native RNA and has the same intervening sequence, and hence are most likely to have the same or very similar RT and PCR efficiencies. DNA standards, such as plasmid DNA and PCR fragments are generally used, assuming that the RT variability is negligible, because the subsequent PCR assays are generally used to calculate PCR efficiency. The use of DNA standards can be an accurate estimation of qRT-PCR efficiency only when there is minimal RT variability. It is worth noting that *in vitro *transcribed RNA from PCR products is not the same as full length mRNA, concerning secondary or tertiary mRNA structures which may affect RT efficiency. Thus, for *in vitro *transcribed RNA, it is important to have sequences upstream and downstream from the amplified sequence identical or similar to the "natural" target. The use of one-step qRT-PCR in this study further reduced the variability of qRT-PCR by lowering the contamination risk because of circumventing the need to open the reaction tubes between RT and PCR steps. To adapt the background of the standards to the native RNA samples, each dilution of the standards was spiked with 20 ng E. coli total RNA consisting of the same amount of nucleic acids as the native RNA samples.

**Figure 2:**
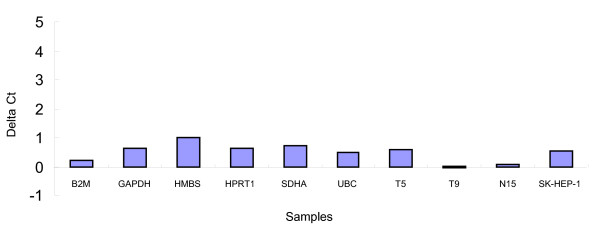
**QRT-PCR inhibitor detection in standards and several RNA samples**. ΔCt for amplification of alien RNA in standards spiked with 20 ng E. coli total RNA and 20 ng RNA from several RNA samples were compared with that of alien RNA alone. The tissue samples contained relatively high and low abundance of qRT-PCR inhibitor existence as determined by the qRT-PCR detection among all samples. Each sample was spiked with 10^5 ^copies of alien RNA transcripts.

Efficiency of qRT-PCR (E) of primer pairs ranged from 87.8% to 94.9%, and correlation coefficients (R^2^) ranged from 0.994 to 0.998 (Table 2). The amplification of alien RNA transcripts among standards and 20 ng native RNA samples was at a similar level, thus indicating analogical qRT-PCR efficiency (Figure 2).

### Expression levels of candidate reference genes

**Figure 3:**
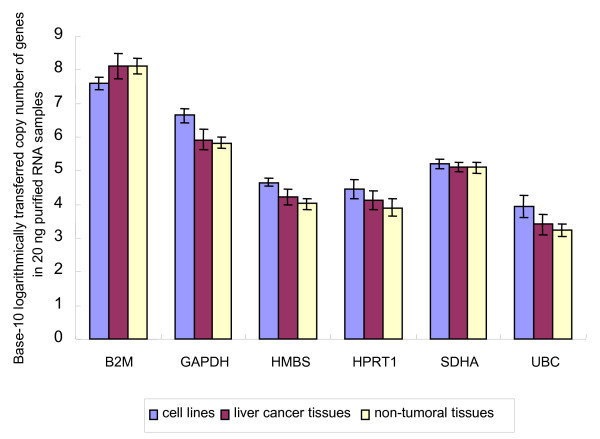
**Expression levels of 6 putative reference genes in different sample panels**. Average copy number of each gene in 20 ng of purified RNA samples was transferred on a base-10 logarithmic scale. An approximately 10,000-fold expression difference is apparent between the most abundant (*B2M*) and the rarest (*UBC*) transcript.

According to the average copy number of each gene in 20 ng purified total RNA of each sample, the six tested genes belong to various abundance classes, with an approximately 10,000-fold expression difference between the most abundant (*B2M*) and the rarest (*UBC*) transcript. The expression levels for individual genes were also different among different sample panels. Four out of six genes showed an expression pattern in cell lines > tumoral tissues > non-tumoral tissues, *SDHA *showed an expression pattern in cell lines > non-tumoral tissues ≈ tumoral tissues, and *B2M *showed an expression pattern in tumoral tissues > non-tumoral tissues > cell lines (Figure 3).

### Expression stability of putative reference genes

The data obtained for each sample and each reference gene were analyzed using both geNorm and NormFinder [1,33]. *GeNorm *provides a ranking of the tested genes based on the reference gene stability measure M which is defined as the average pair-wise variation of a particular gene compared with all other control genes. Genes with higher M values have greater variations of expression. Additionally, the assessment of the pair-wise variations (V_n/n+1_) between each combination of sequential normalization factors allows the identification of the optimal number of reference genes. *NormFinder*, whose strategy is rooted in a mathematical model of gene expression, provides a ranking of the tested genes based on a direct measure of both overall expression variation and the variation between sample subgroups of candidate reference genes. The stability of genes was shown as stability value, and genes with lower stability values have higher expression stability. *NormFinder *has been reported to show relatively low sensitivity towards co-regulation of the candidate normalization genes [33].

**Figure 4:**
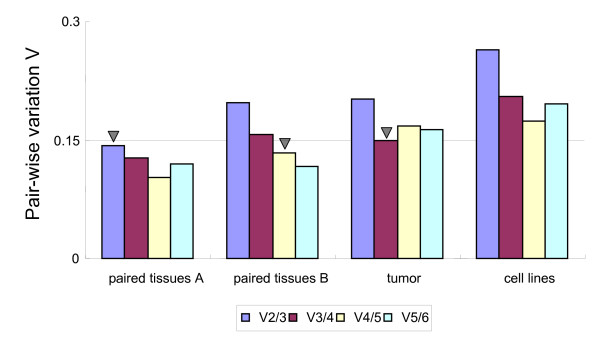
**Determination of the optimal number of reference genes for paired tumoral and non-tumoral tissues, tumor tissues and cell lines separately**. Pair-wise variations (V_n/n+1_) between every combination of sequential normalization factors were calculated to determine the minimum number of reference genes required for accurate normalization in different sample panels (Paired tissues A: paired tumoral and non-tumoral tissues left after the exclusion of RNA samples with relatively high amount of inhibitors; Paired tissues B: all paired tissues). The cutoff value below which the inclusion of an additional reference gene does not result in a significant improvement of normalization, was set at 0.15 (arrowhead = minimum number of reference genes for normalization).

The analysis by *geNorm *suggested that all studied genes reached a high expression stability with low M values (Table 4 and 5), below the default limit of M = 1.5[1]. The optimal reference genes for normalizing gene expression data between paired tumoral and non-tumoral liver tissues were *HMBS *and *GAPDH *(Table 4; Figure 4). *HMBS, GAPDH *and *UBC *were identified to be suitable for the normalization of gene expression data among tumor tissues. The combination of *HMBS, B2M*, *SDHA *and *GAPDH *was suitable for normalizing gene expression data among five liver cancer cell lines, yielding a V_4/5 _value of 0.175, which is close to the cutoff value 0.15. The reason why there was no low enough V value in cell lines may be due to the limited number of cell lines or candidate reference genes included in the evaluation.

**Table 4:** Reference genes ranked in order of increasing expression stability in paired liver tissues*.

**paired tissues A (n = 22)**				**Paired tissues B (n = 32)**			
**geNorm**	**M value**	**NormFinder**	**Stability value**	**geNorm**	**M value**	**NormFinder**	**Stability value**

*B2M*	0.799	*B2M*	0.149	*B2M*	0.826	*UBC*	0.207
*HPRT1*	0.658	*HPRT1*	0.105	*HPRT1*	0.816	*SDHA*	0.203
*UBC*	0.654	*UBC*	0.101	*UBC*	0.771	*HPRT1*	0.164
*SDHA*	0.598	*GAPDH*	0.068	*SDHA*	0.711	*B2M*	0.159
*GAPDH*	0.566	*SDHA*	0.052	*GAPDH*	0.692	*GAPDH*	0.145
*HMBS*	0.514	*HMBS*	0.021	*HMBS*	0.629	*HMBS*	0.084
*HMBS-GAPDH*	0.417	*HMBS-SDHA*	0.033	*HMBS-SDHA*	0.489	*HMBS-B2M*	0.079

Paired tissues A: paired tumoral and non-tumoral tissues left after the exclusion of RNA samples with relatively high amount of inhibitors; Paired tissues B: all paired tissues. *Increases from top to bottom.

**Table 5:** Reference genes ranked in order of increasing expression stability in tumoral tissues and cell lines*.

**Tumoral tissues**				**Cell lines**			
**geNorm**	**M value**	**NormFinder**	**Stability value**	**geNorm**	**M value**	**NormFinder**	**Stability value**

*B2M*	1.091	*B2M*	0.661	*UBC*	1.277	*UBC*	0.791
*SDHA*	0.953	*SDHA*	0.517	*HPRT1*	1.112	*HPRT1*	0.652
*HPRT1*	0.863	*HPRT1*	0.448	*GAPDH*	1.002	*GAPDH*	0.457
*UBC*	0.837	*UBC*	0.393	*SDHA*	0.921	*SDHA*	0.408
*GAPDH*	0.763	*GAPDH*	0.294	*B2M*	0.887	*B2M*	0.321
*HMBS*	0.676	*HMBS*	0.060	*HMBS*	0.857	*HMBS*	0.272
*HMBS-GAPDH*	0.461	-	-	*HMBS-SDHA*	0.620	-	-

* Increases from top to bottom

The results of the analysis by *NormFinder *appeared to be identical to the one determined using *geNorm *in each sample panel, except for the position of two genes with close stability, *GAPDH *and *SDHA*, whose ranking was inverted in paired tissues (Table 4). Interestingly, the stable value of the best combination of two genes determined by *NormFinder *for paired tissues was slightly higher than that of *HMBS *alone (Table 4), indicating *HMBS *alone may be sufficient as reference gene for expression studies in paired tissues.

When comparing the data of the paired tissue group in which 10 tissue RNA samples with high inhibitory substances were excluded, a higher stability of each gene, and a higher similarity between the two algorithms became evident (Table 4; Figure 4), indicating that the existence of qRT-PCR inhibitors in RNA samples can increase the variation of qRT-PCR assay.

It is worth noting that clonal variation that has been described to occur in HCC [34], might induce bias in gene expression studies. While genomic heterogeneity may interfere with gene expression studies aimed at identifying a malignant genotype, this phenomenon should be of less importance for the selection of reference genes which are constitutively expressed and are generally involved in basic functions needed for the sustenance of the cell.

As shown in Table 6, major differences were evident in the ranking order and gene stability of the candidate genes in paired tissues compared to a similar study previously published by Kim et al. [35]. UBC was ranked as the most stably expressed gene in the previous study, but was ranked as the fourth using both *geNorm *and *NormFinder *in our study. In addition, the M values for the candidate reference genes were much lower in our study, thus indicating a high reliability of the data. The notably higher stability of putative reference genes in our study may be due to the following: *(i) *matching tumoral tissue with the non-tumoral tissue from the same patient may have reduced the noise deriving from diversity of gene expression among individuals; (ii) the integrity of RNA was assessed and controlled using Agilent Bioanalyzer; (iii) qRT-PCR inhibitor was maintained at an acceptable level in all standards and samples; and (iv) qRT-PCR efficiency was determined for each primer pair.

**Table 6:** Reference genes ranked in order of increasing expression stability in the current study and the study of Kim et al. [35].

	Current study*		**Study of Kim et al.**	
	
**Rank**	**Genes**	**M value**	**Genes**	**M value**
**6**	*B2M*	0.799	*B2M*	2.60
**5**	*HPRT1*	0.658	*RPL13A*	2.50
**4**	*UBC*	0.654	*GAPDH*	2.43
**3**	*SDHA*	0.598	*HPRT1*	2.26
**2**	*GAPDH*	0.566	*HMBS*	2.18
**1**	*HMBS*	0.514	*UBC*	2.17
	*HMBS-GAPDH*	0.417	*UBC-HMBS*	1.02

* Data shown are from the sample panel of combined tumoral and non-tumoral tissues

## Conclusion

Of six genes studied, *HMBS *was found to be the single best reference gene for expression studies in HCC. The appropriate choice of combination of more than one reference gene to improve qRT-PCR accuracy depends on the kind of liver tissues or cells under investigation. Quantitative assessment and control of qRT-PCR inhibitors using an external RNA control can reduce the variation of qRT-PCR assay and facilitate the evaluation of gene stability. Our results may facilitate the choice of reference genes for expression studies in HCC.

## Abbreviations

qRT-PCR: quantitative reverse transcription polymerase chain reaction; HCC: hepatocellular carcinoma; B2M: beta-2-microglobulin; GAPDH: glyceraldehyde-3-phosphate dehydrogenase; HMBS: hydroxymethylbilane synthase; HPRT1: hypoxanthine guanine phosphoribosyl transferase 1; SDHA: succinate dehydrogenase flavoprotein subunit A; UBC: ubiquitin C; OD: optical density; RIN: RNA integrity number; Ct: threshold cycle; M: gene stability value; NF: normalization factor; HCV: hepatitis C virus; ΔCt: delta threshold cycle.

## Competing interests

The authors declare that they have no competing interests.

## Authors' contributions

VRC and QS designed, performed, and analyzed the research and wrote the paper. GCS and AR participated in the data analysis. GG and SB conceived the study, participated in its design and helped to draft the manuscript. All authors read and approved the final manuscript.

## Pre-publication history

The pre-publication history for this paper can be accessed here:



## Supplementary Material

Additional file 1 **Supplementary table 1–5**. Supplementary table 1–5 (doc format) indicate the sequences of primers for cloning and the length of amplicons, background of cell lines, background of liver tissue samples, copy number of the standards, and RNA quality. Click here for file

Additional file 2 **Melting curve analysis obtained for the *B2M *gene**. Melt curve peak chart (rtf format) collected using the Bio-Rad iQ5 Software 2.0 (Bio-Rad) during calibration experiments of the selected primer pair for the *B2M *gene on an iQ™5 Multicolor Real-Time PCR Detection System (Bio-Rad). RFU: relative fluorescence units; T: temperature. Click here for file

Additional file 3 **Melting curve analysis obtained for the *GAPDH *gene**. Melt curve peak chart (rtf format) collected using the Bio-Rad iQ5 Software 2.0 (Bio-Rad) during calibration experiments of the selected primer pair for the *GAPDH *gene on an iQ™5 Multicolor Real-Time PCR Detection System (Bio-Rad). RFU: relative fluorescence units; T: temperature. Click here for file

Additional file 4 **Melting curve analysis obtained for the *HMBS *gene**. Melt curve peak chart (rtf format) collected using the Bio-Rad iQ5 Software 2.0 (Bio-Rad) during calibration experiments of the selected primer pair for the *HMBS *gene on an iQ™5 Multicolor Real-Time PCR Detection System (Bio-Rad). RFU: relative fluorescence units; T: temperature. Click here for file

Additional file 5 **Melting curve analysis obtained for the *HPRT1 *gene**. Melt curve peak chart (rtf format) collected using the Bio-Rad iQ5 Software 2.0 (Bio-Rad) during calibration experiments of the selected primer pair for the *HPRT1 *gene on an iQ™5 Multicolor Real-Time PCR Detection System (Bio-Rad). RFU: relative fluorescence units; T: temperature. Click here for file

Additional file 6 **Melting curve analysis obtained for the *SDHA *gene**. Melt curve peak chart (rtf format) collected using the Bio-Rad iQ5 Software 2.0 (Bio-Rad) during calibration experiments of the selected primer pair for the *SDHA *gene on an iQ™5 Multicolor Real-Time PCR Detection System (Bio-Rad). RFU: relative fluorescence units; T: temperature. Click here for file

Additional file 7 **Melting curve analysis obtained for the *UBC *gene**. Melt curve peak chart (rtf format) collected using Bio-Rad iQ5 Software 2.0 (Bio-Rad) during calibration experiments of the selected primer pair for the *UBC *gene on an iQ™5 Multicolor Real-Time PCR Detection System (Bio-Rad). RFU: relative fluorescence units; T: temperature. Click here for file

Additional file 8 **PCR amplification chart for *B2M *and *GAPDH *genes in two samples containing genomic DNA**. PCR amplification chart (rtf format) collected using Bio-Rad iQ5 Software 2.0 (Bio-Rad) during calibration experiments of the selected primer pair for the *B2M *and *GAPDH *genes on an iQ™5 Multicolor Real-Time PCR Detection System (Bio-Rad) in two samples still contained genomic DNA as shown in previous RT negative controls. The Ct values of RT negative reactions were 16.37–17.94 lower than that of RT positive control. A: RT positive reaction. B: RT negative reaction for the same samples. RFU: relative fluorescence units; T: temperature. Click here for file
