# Supplementary material for: Validation of putative reference genes for gene expression studies in human hepatocellular carcinoma using real-time quantitative RT-PCR
Source: BMC Cancer. 2008 Nov 27;8:350. doi: 10.1186/1471-2407-8-350 (PMC2607287; doi:10.1186/1471-2407-8-350)
Supplement: Additional file 1 — Supplementary table 1–5. Supplementary table 1–5 (doc format) indicate the sequences of primers for cloning and the length of amplicons, background of cell lines, background of liver tissue samples, copy number of the standards, and RNA quality. [file 1471-2407-8-350-S1.doc]

**Supplementary table 1: Details of primers for PCR cloning of each of the 6 evaluated genes.**

| Gene | Primer | Sequence (5’→3’) | Amplicon Length |
| --- | --- | --- | --- |
| *B2M* | Forward | TGAAGCTGACAGCATTCGG | 131 bp |
|  | Reverse | CTGCTGGATGACGTGAGTAAA |  |
| *GAPDH* | Forward | TGGGTGTGAACCATGAGAAG | 472 bp |
|  | Reverse | GTGTCGCTGTTGAAGTCAGA |  |
| *HMBS* | Forward | ATGTCTGGTAACGGCAATGC | 179bp |
|  | Reverse | CCTGTGGTGGACATAGCAATGA |  |
| *HPRT1* | Forward | TGTAGCCCTCTGTGTGCTCAAG | 523 bp |
|  | Reverse | GGCGATGTCAATAGGACTCCAG |  |
| *SDHA* | Forward | CCCGAGGTTTTCACTTCACTGT | 268 bp |
|  | Reverse | CCAGTTGTCCTCCTCCATGTTC |  |
| *UBC* | Forward | CGGTGAACGCCGATGATTAT | 124 bp |
|  | Reverse | ATCTGCATTGTCAAGTGACGA |  |

**Supplementary table 2: Backgrounds of cell lines used.**

| **Cell lines** | **Aliases** | **ATCC**  **Number** | **Source** | | | | |
| --- | --- | --- | --- | --- | --- | --- | --- |
|  |  |  | **Organ** | **Tumor**  **entity** | **Age**  **(years)** | **Gender** | **Ethnicity** |
| Hep 3B2.1-7* | Hep-3B;  Hep3B | HB-8064 | liver | HCC | 15 | male | Caucasian |
| HepG2 |  | HB-8065 | liver | HCC | 8 | male | Black |
| HUH7 | JTC-39 |  | liver | HCC  (well differentiated) | 57 | male | Asian |
| SK-HEP-1 |  | HTB-52 | liver | adenocarcinoma | 52 | male | Caucasian |
| SNU-182* |  | CRL-2235 | liver | HCC | 24 | male | Asian |

*****contains hepatitis B virus genome

**Supplementary table 3: Backgrounds of liver tissues used.**

| **Code no.** | **Gender** | **Age**  **(years)** | **Tumoral**  **tissue** | **Non-tumoral**  **tissue** | **Code no.** | **Gender** | **Age**  **(years)** | **Tumoral**  **tissue** | **Non-tumoral**  **tissue** |
| --- | --- | --- | --- | --- | --- | --- | --- | --- | --- |
| 1 | M | 66 | HCC | Normal | 11 | M | 76 | HCC | Normal |
| 2 | M | 70 | HCC | Normal | 12 | M | 72 | HCC | N.A. |
| 3 | F | 79 | HCC | Normal | 13 | F | 72 | HCC | Cirrhosis |
| 4 | M | 77 | HCC | Normal | 14 | F | 68 | HCC | Hepatitis B, cirrhosis |
| 5 | M | 48 | HCC | Normal | 15 | M | 60 | HCC | Hepatitis C |
| 6 | F | 60 | HCC | Hepatitis C, cirrhosis | 16 | M | 69 | N.A. | Hepatitis C, cirrhosis |
| 7 | M | 70 | HCC | Normal | 17 | M | 75 | N.A. | Normal |
| 8 | F | 80 | HCC | Hepatitis C, cirrhosis | 18 | M | 70 | HCC | N.A. |
| 9 | M | 46 | HCC | Normal | 19 | M | 59 | HCC | N.A. |
| 10 | F | 45 | HCC | Cirrhosis | 20 | M | 45 | HCC | Hepatitis B, cirrhosis |

*****RNA from Ambion.Inc. HCC: Hepatocellular carcinoma. M: male. F: female. N.A.: not available

**Supplementary table 4: Range of copy numbers of standards.**

| **Standards** | **Copy number** |
| --- | --- |
| *B2M* | 10^9^-10^5^ |
| *GAPDH* | 10^8^-10^4^ |
| *HMBS* | 10^7^-10^3^ |
| *HPRT1* | 10^7^-10^3^ |
| *SDHA* | 10^8^-10^4^ |
| *UBC* | 10^7^-10^3^ |

**Supplementary table 5: Details of RNA quality.**

| Sample | OD260/280 | OD260/230 | RIN | Sample | OD260/280 | OD260/230 | RIN |
| --- | --- | --- | --- | --- | --- | --- | --- |
| Hep 3B | 2.09 | 1.9 | 10 | T9 | 2.1 | 0.71 | 9.5 |
| HepG2 | 2.08 | 2.08 | 10 | N9 | 2.1 | 1.32 | 9.1 |
| HUH7 | 2.06 | 1.65 | 10 | T10 | 2.08 | 1.49 | 9.7 |
| SK-HEP-1 | 2.05 | 2.09 | 10 | N10 | 2.12 | 1.03 | 8.8 |
| SNU-182 | 2.07 | 1.09 | 10 | T11 | 1.85 | 0.81 | 8.8 |
| T1 | 2.08 | 2.13 | 7.1 | N11 | 2.08 | 1.5 | 9.1 |
| N1 | 2.07 | 2.06 | 9.5 | T12 | 2.13 | 1.11 | 9.6 |
| T2 | 2.1 | 0.78 | 7.7 | T13 | 2.08 | 1.34 | 9.3 |
| N2 | 2.11 | 1.34 | 6.5 | N13 | 2.08 | 1.96 | 9.2 |
| T3 | 2.11 | 1.98 | 7.5 | T14 | 2.12 | 0.71 | 9.2 |
| N3 | 2.11 | 2.23 | 8.2 | N14 | 2.09 | 1.05 | 9.4 |
| T4 | 2.08 | 1.87 | 9 | T15 | 2.07 | 0.43 | 9.6 |
| N4 | 2.1 | 1.74 | 9.3 | N15 | 2.08 | 1.69 | 8.9 |
| T5 | 2.13 | 1.24 | 9.1 | N16 | 2.09 | 1.19 | 8.1 |
| N5 | 2.17 | 1.18 | 9.5 | T17 | 2.08 | 1.43 | 9 |
| T6 | 2.03 | 1.17 | 7.1 | N17 | 2.06 | 1.49 | 8.5 |
| N6 | 2.05 | 1.97 | 9.4 | T18 | 2.1 | 2.03 | 9 |
| T7 | 2.05 | 1.03 | 9.8 | T19 | 2.09 | 1.71 | 8.6 |
| N7 | 2.05 | 2.05 | 9.7 | T20 | 2.07 | 1.87 | 8.9 |
| T8 | 2.06 | 1.64 | 9.7 | N20 | 2.03 | 1.25 | 9.2 |
| N8 | 2.03 | 1.24 | 9.1 |  |  |  |  |

*****RNA from Ambion, Inc. OD260/280: the ratio of optical density (OD) between wavelength of 260 nm and that of 280 nm. OD260/230: the ratio of optical density (OD) between wavelength of 260 nm and that of 230 nm. RIN: RNA integrity number.
