# Supplementary material for: Validation of putative reference genes for gene expression studies in human hepatocellular carcinoma using real-time quantitative RT-PCR
Source: BMC Cancer. 2008 Nov 27;8:350. doi: 10.1186/1471-2407-8-350 (PMC2607287; doi:10.1186/1471-2407-8-350)
Supplement: Additional file 6 — Melting curve analysis obtained for the SDHA gene. Melt curve peak chart (rtf format) collected using the Bio-Rad iQ5 Software 2.0 (Bio-Rad) during calibration experiments of the selected primer pair for the SDHA gene on an iQ™5 Multicolor Real-Time PCR Detection System (Bio-Rad). RFU: relative fluorescence units; T: temperature. [file 1471-2407-8-350-S6.rtf]

Additional file 6: Melting curve analysis obtained for the SDHA gene.
