# Supplementary material for: Validation of putative reference genes for gene expression studies in human hepatocellular carcinoma using real-time quantitative RT-PCR
Source: BMC Cancer. 2008 Nov 27;8:350. doi: 10.1186/1471-2407-8-350 (PMC2607287; doi:10.1186/1471-2407-8-350)
Supplement: Additional file 8 — PCR amplification chart for B2M and GAPDH genes in two samples containing genomic DNA. PCR amplification chart (rtf format) collected using Bio-Rad iQ5 Software 2.0 (Bio-Rad) during calibration experiments of the selected primer pair for the B2M and GAPDH genes on an iQ™5 Multicolor Real-Time PCR Detection System (Bio-Rad) in two samples still contained genomic DNA as shown in previous RT negative controls. The Ct values of RT negative reactions were 16.37–17.94 lower than that of RT positive control. A: RT positive reaction. B: RT negative reaction for the same samples. RFU: relative fluorescence units; T: temperature. [file 1471-2407-8-350-S8.rtf]

Additional file 8: PCR amplification chart for B2M and GAPDH genes in two samples containing genomic DNA.
